# The trochlear sulcus of the native knee is consistently orientated close to the sagittal plane despite variation in distal condylar anatomy

**DOI:** 10.1007/s00167-021-06667-9

**Published:** 2021-07-21

**Authors:** Ilya Borukhov, Christina I. Esposito, Petros Ismailidis, Sally LiArno, Jenna P. Lyon, Thomas F. McCarthy, Peter McEwen

**Affiliations:** 1 Stryker, Mahwah, NJ USA; 2 Stryker, Sydney, NSW Australia; 3 The Orthopaedic Research Institute of Queensland (ORIQL), 7 Turner St, Pimlico Townsville, QLD Australia; 4 Department of Orthopaedics and Traumatology, University Hospital of Basel, Spitalstrasse 21, 4031 Basel, Switzerland

**Keywords:** TKA, Total knee arthroplasty, Distal trochlear orientation, Femoral component design, mLDFA, Trochlear sulcus anatomy

## Abstract

**Purpose:**

The aim of this study was to describe the native trochlear orientation of non-arthritic knees in three planes and to quantify the relationship between trochlear and distal condylar anatomy across race and sex.

**Methods:**

Computed tomography scans of 1578 femora were included in this study. The mediolateral position of the trochlear sulcus, the distal trochlear sulcus angle (DTSA) the medial sulcus angle (MSA) and the lateral sulcus angle (LSA) as well as the mechanical lateral distal femoral angle (mLDFA) were measured relative to a standard reference coordinate system. Multiple linear regression analyses were performed to account for potential confounding variables.

**Results:**

The mediolateral position of the trochlear sulcus had minimal mean deviation of the sagittal femoral plane. The mean DTSA was 86.1° (SD 2.2°). Multilinear regression analysis found mLDFA, sex, and age all influence DTSA (*p* < 0.05), with mLDFA having by far the greatest influence (*r*^2^ = 0.55). The medial facet of the trochlear sulcus was found to be flat proximally and more prominent distally. The lateral facet was relatively uniform throughout the arc.

**Conclusion:**

In non-arthritic knees, due to a strong positive correlation between the DTSA and the mLDFA, the trochlear sulcus is consistently orientated in the sagittal femoral plane regardless of distal condylar anatomy. Minor deviations from the sagittal plane occur in a lateral direction in the middle part and in a medial direction at the proximal and distal part of the trochlea. These findings have relevance regarding the biomimetic design of total knee implants.

## Introduction

Femoral component design in total knee arthroplasty (TKA) has evolved to better reflect normal anatomy and encourage optimal biomechanics. The biomimicry evident in femoral condylar design is, however, generally not reflected in the trochlea. The native trochlear sulcus is orientated almost parallel to the sagittal plane of the femur but lateral to the midline, whereas in conventional TKA design the sulcus has a valgus orientation that tracks distally to the midpoint between the condyles [1, 2]. The sagittal plane orientation of the native trochlea positions the extensor mechanism for maximum mechanical advantage, whereas the typical prosthetic trochlea is oriented to facilitate a soft capture of the patella with a widened sulcus angle and lowered lateral trochlea to reduce lateral retinacular tension, lateral shear, and compressive loads on the patella [3].

Femoral component placement in kinematic alignment (KA) technique aims to restore the pre-arthritic joint line through matched distal and posterior femoral resections accounting for cartilage loss [4]. This placement algorithm necessarily aligns the femoral component along the pre-arthritic mechanical Lateral Distal Femoral Angle (mLDFA), as defined by the angle between the mechanical axis of the femur and the distal joint line in the coronal plane, rather than perpendicular to the mechanical axis of the femur. The pre-arthritic mLDFA defines not only the orientation of the distal condyles but the orientation of the trochlea and therefore the relationship between the mLDFA and the sulcus is key to understanding how well a TKA replicates the native trochlea being replaced.

Previous work has defined the relationship between the trochlear sulcus and the sagittal plane and long axes of the femur [1]. Cadaveric studies and advanced 3D imaging has improved our understanding of the patellofemoral articulation [5, 6]. A deepened trochlear sulcus with a lateral wall build-up appears to improve patellar tracking, tilt, and contact pressures [7, 8]. However, the great variation of the patellofemoral sulcus design among the individual prosthesis manufacturers and continued patellofemoral dislocations underscores the need for further evaluation of medial and lateral sulcus angles. More recent work based on a relatively small sample size has also demonstrated that neither mechanical alignment (MA) or KA techniques are biomimetic in terms of sulcus orientation or lateral trochlear height, although KA produces a sulcus orientation less deviant from normal [9]. However, the relationship between the trochlea and the mLDFA has not been studied in-depth, although it has implications concerning KA and prosthetic design. We, therefore, undertook a computerized tomography (CT) based study with a large sample size to examine the relationship between mLDFA and trochlea orientation.

The purpose of this study was to describe the trochlear orientation as well as the medial and lateral sulcus angles (MSA and LSA) and quantify the relationship between these factors and the distal condylar anatomy across race and sex in non-arthritic knees. We hypothesized that the mLDFA would correlate with the trochlear orientation.

## Methods

### Biomorphometric CT database

A CT-scan-based modelling and analytics system, composed of scans of over 25,000 bone segments was used for this study (SOMA, Stryker, Mahwah, NJ) [10]. This study was done in agreement with the ethical standards of the 1964 Helsinki declaration and its later amendments. All scans were obtained per local legal and regulatory requirements which included ethics board approval and informed patient consent, where appropriate. CT scans were acquired exclusively for medical indications such as polytrauma (20%), CT angiography (70%), and other reasons (10%, i.e. total joint replacement). Out of 2890 lower extremity CT scans screened, 1578 femora were included in this study. Inclusion criteria were the presence of CT scan of the entire femur. Exclusion criteria were signs of degenerative arthritis, id est presence of osteophytes as described by the Kellgren and Lawrence classification system [11], bone deformities, or evidence of previous surgery. Patient demographics for the femora included in this study are shown in Table 1.

**Table 1:** Sample size and subject demographics included in this study

All	1578	Age (years)	64 ± 16 (12–109)
Caucasian	1128		
Male	589	Height (m)	1.67 ± 0.11 (1.33–1.99)
Female	539		
Asian	450	Body mass (kg)	71 ± 18 (29–181)
Male	178		
Female	272	BMI (kg/m^2^)	25.1 ± 5.2 (13.3–54.6)
Bilateral	554		

Continuous variables represented as mean ± SD (range)

*BMI* body mass index

### Trochlear sulcus morphological measurements

A CT computer-aided analysis software (SOMA, Stryker Anatomy Analysis Tool, Stryker, Mahwah, NJ) was used to segment bone surfaces from the CT images and create constructions on a correspondence bone, using predefined landmarks and user-defined points, which were then mapped onto each individual subject for analysis. This method produces reproducible and consistent constructs for each specimen, shown to have a margin of error of < 2 mm and < 1° and a demonstrated measurement variation of 0.2%, typically less than that of interobserver error [12, 13].

**Fig. 1:**
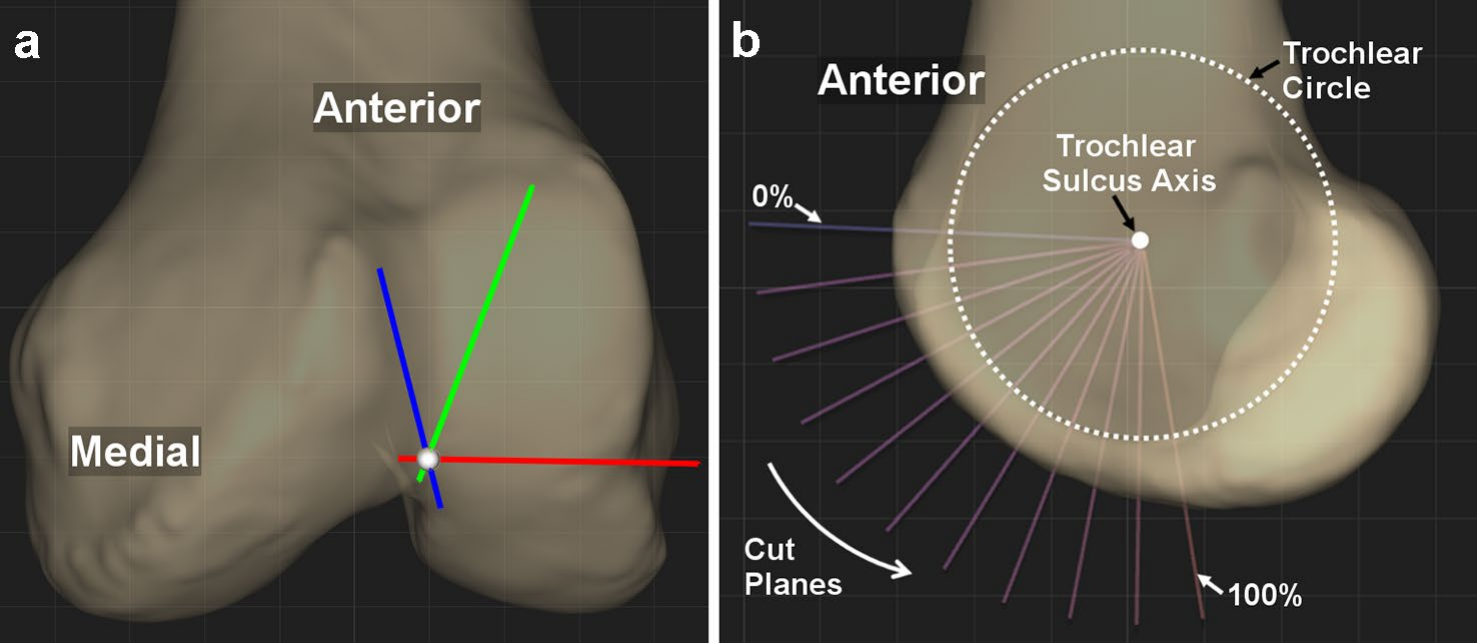
Constructions on the left femur of a representative CT bone model. **A** Coordinate system established on each femur, with + Z (blue) pointing superior along the mechanical axis, + Y (green) pointing anterior perpendicular to the surgical transepicondylar axis, + X (red) pointing lateral, centred at the apex of the intercondylar notch. **B** The trochlear sulcus axis was established as the line crossing the centre of this circle and parallel to the cylindrical axis. A total of eleven coaxial cutting planes rotated equally about the trochlear sulcus axis were created. Each coaxial cutting plane was defined by the percentage along the arc of the trochlear sulcus with 0% and 100% intersecting the most anteroproximal and posterodistal points, respectively

Before measurements were taken, each femur was aligned to a standard reference coordinate system, similar to that described by Grood and Suntay [14] varying in the rotation of the coronal plane (Fig. 1A). The apex of the intercondylar notch of the knee was considered the origin of the coordinate system. The femoral mechanical axis was defined as the line passing through the centre of the femoral head and the apex of the intercondylar notch at the knee. The coronal plane contained the mechanical axis and was parallel to the surgical transepicondylar axis, a reference commonly used in total knee arthroplasty [15]. The sagittal plane was perpendicular to both the transverse and coronal planes.

**Fig. 2:**
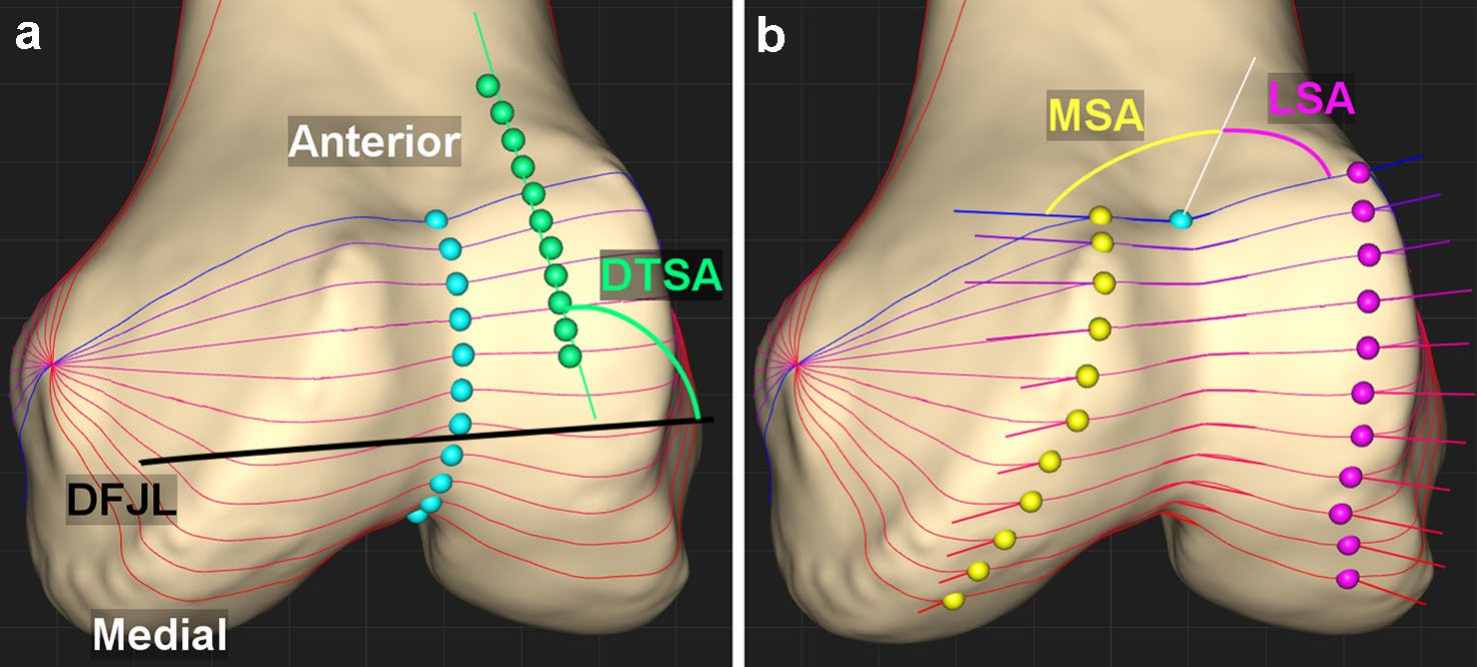
**A** Trochlear dwell points (light blue), trochlear roll-out projection points (light green). The black line is the distal femoral joint line (DFJL), defined as the line connecting the most distal points on the medial and lateral femoral condyles when viewed in the coronal plane. The angle shown is the distal trochlear sulcus angle (DTSA, light green). **B** Trochlear sulcus angle measured at each cross-section with medial peaks (yellow) and lateral peaks (purple). The angle shown is the sulcus angle at the most anteroproximal cross section. To evaluate the medial and lateral facets of the trochleas, the femoral medial sulcus angle (MSA) and the lateral sulcus angle (LSA) were measured at each of the eleven coaxial cutting planes

The trochlear sulcus was characterized according to previously described methods [5, 6, 16, 17] (Fig. 1B). A circle was best fit to the trochlear sulcus in the sagittal plane (Fig. 1B). The trochlear sulcus axis was established as the line crossing the centre of this circle and parallel to the cylindrical axis (Fig. 1B). A total of eleven coaxial cutting planes rotated equally about the trochlear sulcus axis were created (Fig. 1B). Each coaxial cutting plane was defined by the percentage along the arc of the trochlear sulcus with 0% and 100% intersecting the most anteroproximal and posterodistal points, respectively (Fig. 1B). The deepest point of the trochlear sulcus was found at the cross-section of each cutting plane. The connection of the deepest point of each cross section was collectively defined by the sulcus (Fig. 2A). Each point was then transferred to the coronal plane using a roll-out projection (Fig. 2A), as described previously in the literature [5, 6, 17]. With this process, the distance between points in the sagittal plane as well as the mediolateral distance of each point to the sagittal plane was maintained (Fig. 2A).

**Fig. 3:**
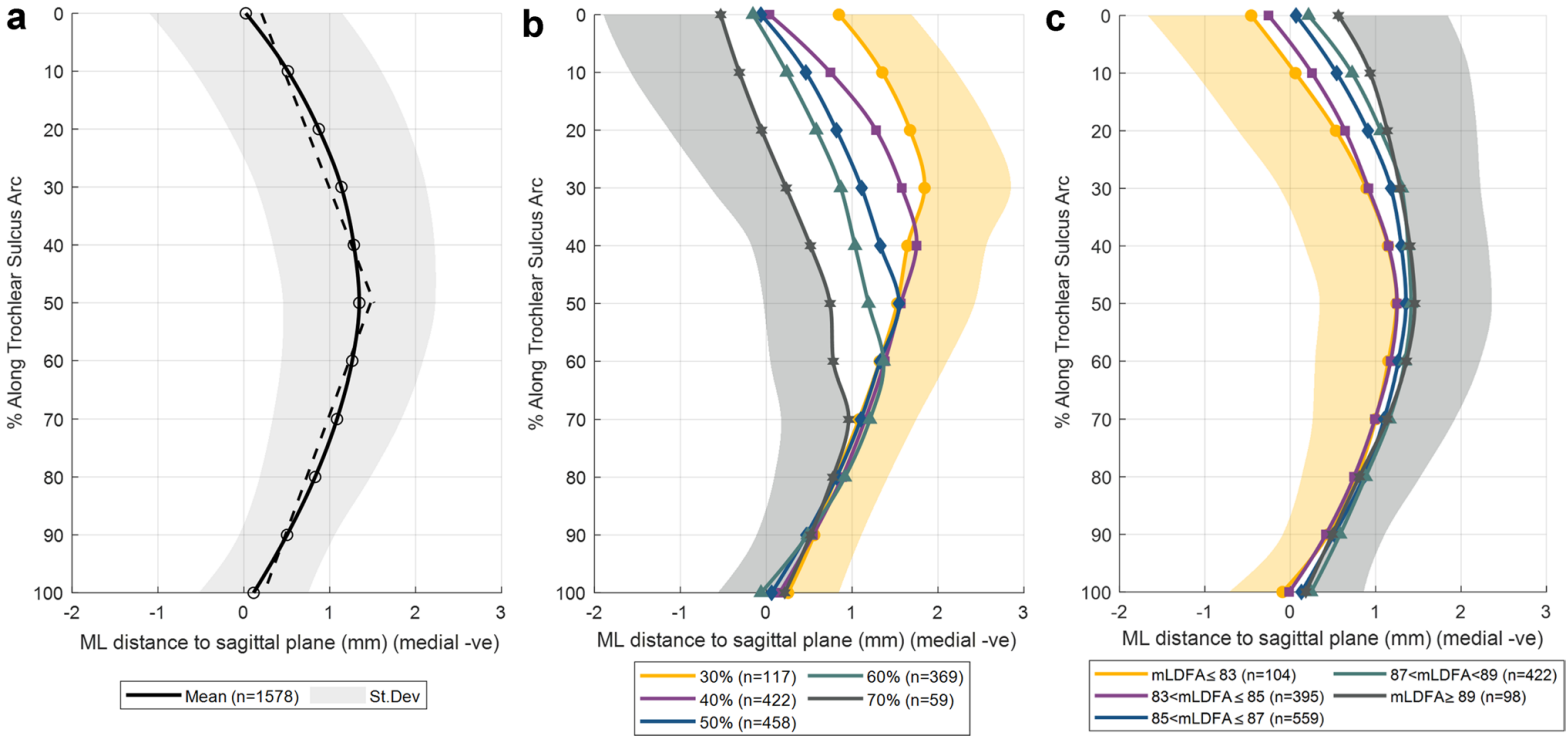
Mediolateral position of the dwell points along the trochlear sulcus arc, with 0% being the most anteroproximal point. **A** The mean (solid line) and one standard deviation (shaded region) mediolateral distance from the deepest point of the trochlear sulcus to the sagittal plane (negative values represent medial) along the trochlea sulcus. Dotted lines indicate the bilinear approximation for the mean. The inflection point of the trochlear sulcus was defined as the point separating the proximal and distal linear approximations. **B** Mean of subgroups separated by inflection point location, expressed as percentage along the arc of the trochlear sulcus with 0% and 100% being the most anteroproximal and posterodistal points, respectively. For clarity, the lower and upper standard deviations of the 70% and 30% subgroups, respectively, are illustrated. **C** Mean of subgroups separated by mLDFA. For clarity, the lower and upper standard deviations of the mLDFA ≤ 83 and mLDFA > 89 subgroups, respectively, are illustrated

We measured the mediolateral distance from the trochlear sulcus to the sagittal plane. The inflection point of the trochlear sulcus was defined as the point separating the proximal and distal linear approximations and expressed as a percentage along the arc of the trochlear sulcus. It was determined using a bilinear approximation to best fit two linear functions to the trochlear sulcus roll-out points (Fig. 3A). The slope of the proximal and distal portions was defined as mm position per % travelled along the trochlear sulcus arc and measured along with the root mean square error of the approximation relative to the defined roll-out trochlear sulcus points. We followed the methods previously described [5, 17] using a custom MATLAB script (MATLAB 2019a, The MathWorks, Inc., Natick, MA, USA). To evaluate the medial and lateral facets of the trochlea, the femoral MSA and LSA were measured at each of the eleven coaxial cutting planes. MSA and LSA were measured as the angles between the lines connecting the trochlear dwell point and the medial and lateral femoral condylar peaks respectively, relative to the anterior–posterior axis of the reference coordinate system (Fig. 2B). The sum of MSA and LSA represents the trochlea sulcus angle at a given cut plane.

Furthermore, the distal femoral joint line (DFJL) was measured to determine the distal trochlear sulcus angle (DTSA) and the mLDFA. The DFJL was defined as the line connecting the most distal points on the medial and lateral femoral condyles when viewed in the coronal plane. The DTSA was defined as the angle between the DFJL and a straight line fitted through the roll-out points previously described. The mLDFA was defined as the angle between the DFJL and the femoral mechanical axis in the coronal plane.

### Statistical methods

Statistical analysis was performed using Minitab software (v19.2; Minitab, LLC, State College, PA, USA). Means and standard deviations were determined for each of the measurements made for the population as a whole and for subpopulations, based on sex and ethnicity. Normality was established with the Anderson–Darling test and assessed graphically. Univariate analysis was performed using t-tests to estimate a difference between groups and subgroups. Pearson’s coefficients were calculated to examine correlations among specimen radiographic measurements and demographic data. Where applicable, multiple linear regression analyses were performed to account for any potential confounding variables, with age, sex, ethnicity, height, body mass, BMI, laterality, and mLDFA being set as independent variables. Significance was set at *p* ≤ 0.05.

## Results

### Distal trochlear sulcus angle (DTSA)

**Fig. 4:**
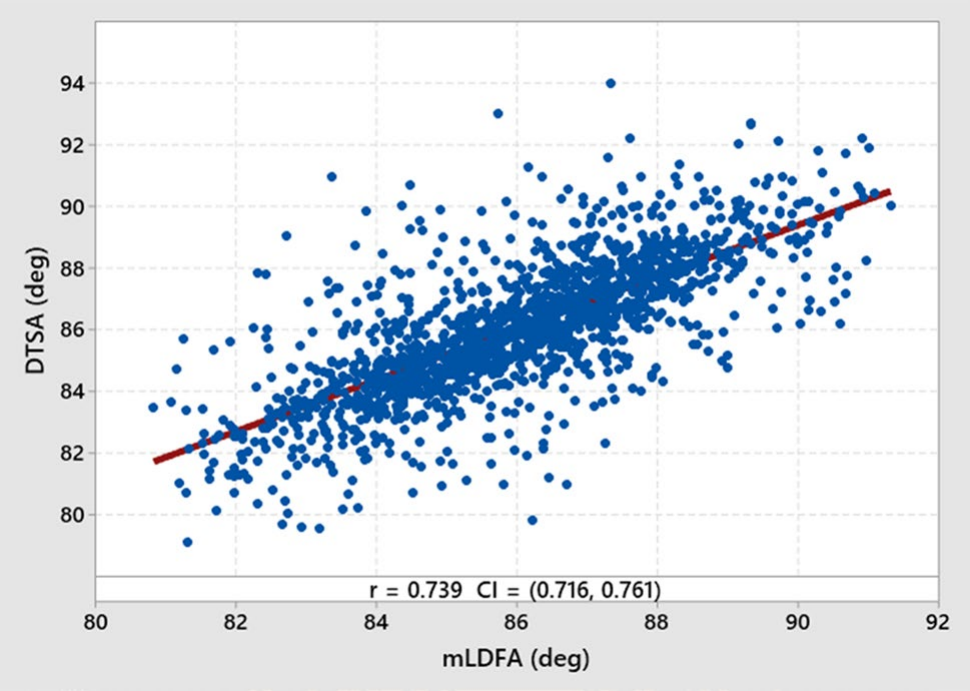
Scatter plot (blue) and regression line (red) of the relationship between distal trochlear sulcus angle (DTSA) and mechanical lateral distal femoral angle (mLDFA). Pearson correlation coefficient of 0.739

The mean DTSA for all femora was 86.1° (SD 2.2°). Table 2 shows the means of morphological measurements across race, sex, and limb side. Ethnicity and sex differences of DTSA can be seen, with Asians having more valgus sulcus orientations than Caucasians (85.8, SD 2.4° vs. 86.2, SD 2.1°, *p* = 0.006), and females having more valgus sulcus orientations than males (85.8, SD 2.3° vs. 86.3, SD 2.0°, *p* < 0.001). Multilinear regression analysis found mLDFA, sex, and age all influence DTSA (*p* < 0.05; Table 3), with mLDFA having by far the greatest influence (*r*^2^ = 0.55). DTSA had a strong positive correlation to mLDFA, with a Pearson correlation coefficient of 0.739 (Fig. 4). Lastly, the mediolateral position of the trochlear sulcus dwell points is also influenced by mLDFA with higher valgus femora having more medially positioned sulcus (Fig. 3C).

**Table 2:** mLDFA and DTSA values of the population as a whole and subgroups. All measurements are in degrees and represented as mean ± SD (range)

	Overall	Female	Male	*P* value	Caucasian	Asian	*P* value	Left	Right	*P* value*
mLDFA	86.0 ± 1.9 (80.8–91.3)	85.6 ± 2.0 (80.8–91.3)	86.5 ± 1.7 (81.1–91.1)	< 0.001	86.1 ± 1.9 (80.8–91.1)	85.8 ± 2.1 (81.1– 91.3)	0.058	86.1 ± 1.9 (81.3–91.1)	85.8 ± 1.8 (80.8–90.9)	0.062
DTSA	86.1 ± 2.2 (79.1–94.0)	85.8 ± 2.3 (79.1–94.0)	86.3 ± 2.0 (79.6–93.0)	< 0.001	86.2 ± 2.1 (79.6–93)	85.8 ± 2.4 (79.1–84.0)	0.006	86.8 ± 1.8 (82–91)	85.8 ± 2.0 (79.6–92.2)	0.016

*mLDFA* mechanical lateral distal femoral angle

*DTSA* distal trochlear sulcus angle

*Calculated with Student *t* test

**Table 3:** Results of multiple linear regression analysis regarding factors influencing the distal trochlear sulcus angle (DTSA)

Factors	*r*^2^	*p* value
mLDFA	0.55	** < 0.001**
Sex	0.01	** < 0.001**
Age	< 0.01	** < 0.001**
BMI	< 0.01	0.938
Height	< 0.01	0.361
Body mass	< 0.01	0.584
Ethnicity	< 0.01	0.103
Laterality	< 0.01	0.269

*mLDFA* mechanical lateral distal femoral angle, *BMI* body mass index

Statistically significant *p*values are identified with bold format

### Mediolateral position and orientation of the trochlear sulcus

Measurement of the mediolateral distance from the trochlear sulcus to the sagittal plane in cross sections along the arc of the trochlear sulcus showed that the trochlear sulcus had only minimal mean deviation from the sagittal plane. The most medial position of the sulcus was found at the proximal and distal ends of the sulcus with a mean deviation from the sagittal plane of 0 mm (SD 1.1 mm) and 0.1 mm (SD ± 0.6 mm), respectively (positive values indicate lateral deviation). Lateral deviation was maximal at the inflection point (1.3 ± 0.9 mm at 50% of the trochlear sulcus arc) (Fig. 3A). The detailed orientation of the trochlear sulcus was lateral on the proximal portion with a slope of 0.5 mm/% (SD 0.4 mm/%) and medial in the distal portion with a slope of − 0.5 mm/% (SD 0.4 mm/%). The root mean square errors for both proximal and distal portions were 0.3 mm (SD 0.1 mm).

The majority of the femora studied had an inflection point at 40% (*n* = 422), 50% (*n* = 458), or 60% (*n* = 369) along the trochlear sulcus arc (Fig. 3B). There was some variability, however, with 151 femora having an inflection point less than 40% and 91 femora having an inflection point greater than 60%. In addition, 87 femora had no discernible inflection point as their trochlear sulcus sloped laterally (*n* = 47) or medially (*n* = 40) through the entire arc. Furthermore, there was large variability in the mediolateral position of the dwell points, particularly at proximal cross sections, that also varied with inflection point location (Fig. 3A, B).

### MSA, LSA

**Fig. 5:**
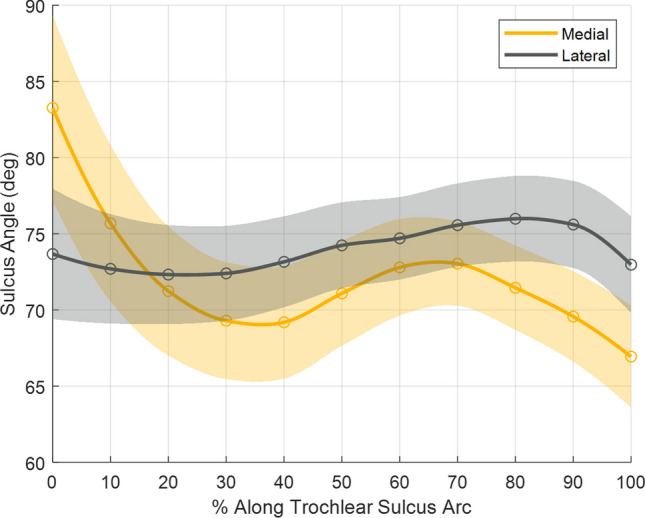
Medial and lateral sulcus angle as a function of percent travel along trochlear sulcus arc. Solid lines indicate mean, shaded area indicates standard deviation

There was a difference in medial and lateral sulcus angles along the trochlear sulcus. The medial facet of the trochlear sulcus is flat proximally (MSA 83.3, SD 6.1° at 0%) and becomes more prominent distally (MSA 71.1, SD 3.5° at 50%). In contrast, the lateral facet is relatively uniform throughout the arc (LSA 74.2, SD 2.8° at 50%, Fig. 5). There were statistically significant differences between medial and lateral sulcus angles (*p* < 0.05) along the entire trochlear sulcus arc, with the largest differences occurring at 0% (Δ9.6°, 95% CI [9.3°, 9.9°]) and 100% (Δ − 6.0°, 95% CI [− 6.3°,− 5.8°]). Regression analysis of the sulcus angles with the continuous and categorical variables measured in this study revealed very weak correlations (*r* < 0.1).

## Discussion

The most important finding of our study is the strong positive correlation between the mLDFA and the DTSA. As the mLDFA decreases (valgus distal femoral orientation) so does the DTSA so that the entire trochlear sulcus is consistently orientated close to the sagittal plane of the femur despite variations in distal femoral condylar orientation. This has implications for prosthetic design and TKA alignment. Furthermore, the multiple regression model has unequivocally demonstrated that the mLDFA (*r*^2^ = 0.55), as opposed to sex or ethnicity, is responsible for almost all the variation in DTSA.

Maillot et al. [16] performed native trochlear sulcus assessment in 58 low-grade arthritic knees and could not identify a correlation between the trochlear sulcus orientation and the hip knee ankle angle (HKAA), mLDFA or mechanical proximal tibial angle. However, they found a correlation between frontal sulcus orientation and mLDFA for the varus sub-group (HKAA < 180°). The authors acknowledged, that the limited number of knees included in their study, may be responsible for the lack of power for some correlations. This may explain the difference in our results.

### Mediolateral position of the trochlear sulcus

The present study found the trochlear sulcus to be orientated medial to lateral in the proximal portion and lateral to medial at the distal portion with a mean and modal inflection point halfway along the sulcus (Fig. 3). That is in agreement with Barink et al. [5] and Chen et al. [6]who also demonstrated bilinear morphology of the sulcus with a variable inflection point. This is not replicated in TKA design in which the patella is driven from lateral to medial through the entire length of the prosthetic sulcus.

### Implications in TKA design

This study has great relevance for the prosthetic design of TKA. Currently, TKA prostheses have a relatively consistent trochlear sulcus design. The typical femoral component has a DTSA of around 83°–85° (5°–7° valgus to the sagittal plane of the component) [18]. Interestingly, most implant manufacturers do not include information about the orientation of the prosthetic trochlea in the surgical technique and prosthesis design rational brochures. Dejour et al. [19] analysed the anatomy of 14 different TKA models and found a valgus orientation of the trochlear sulcus (3.3°–11.7°) in 13 models and vertical orientation in one model. Patellofemoral complications including patellar impingement on prosthetic trochlea, loosening, subluxation or dislocation, fracture, maltracking, and pain, account for about 10% of all TKA complications [20]. These complications suggest that a single trochlear design or implant position is not a universal solution.

**Fig. 6:**
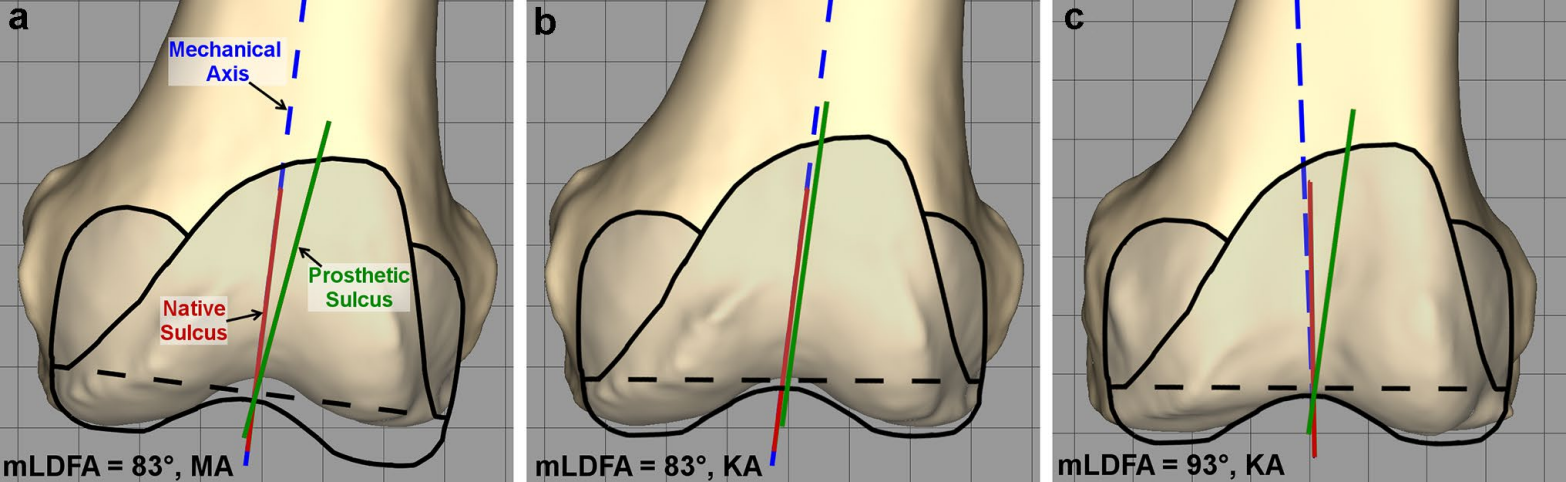
Example of a femoral component with a built-in DTSA of 83° (most common TKA design) implanted in femora with different mLDFA, demonstrating the relevance of the findings of this study. **A** Femoral component implanted with an MA technique. This produces, on average, a prosthetic sulcus (green line) orientated approximately 7° to valgus from native sulcus (red line) regardless of native mLDFA (on this figure, the native femur has an mLDFA of 83°). **B** Femoral component implanted using a KA technique to a femur with a native mLDFA of 83° (severe valgus distal femur). The prosthetic sulcus (green line), on average, closely matches the native sulcus (red line). **C** Femoral component implanted using a KA technique to a femur with a native mLDFA of 93° (varus distal femur). The prosthetic sulcus (green line) deviates, on average, from the native sulcus (red line) 10° to valgus

Furthermore, this study has great relevance for the implant positioning technique. Depending on the native mLDFA, the DTSA of the implant and the implant positioning technique applied, large deviations between native and prosthetic sulcus orientations are possible. Figure 6 presents an example of a femoral component with a built-in DTSA of 83° (most common TKA design) implanted in femora with different mLDFA, demonstrating the relevance of the findings of this study. The primary restraint to lateral patellar translation in the native knee is the lateral trochlea. There is evidence that this is not consistently restored with total knee arthroplasty and that upsizing the femoral component to restore lateral trochlea height will overstuff elsewhere and result in mediolateral overhang [4]. A biomimetic orientation of the trochlea, as in the latter example above, without restoration of lateral trochlea height, could potentially provoke patellar instability. This could potentially be accentuated by flexion of the femoral component which may delay capture of the patella in the prosthetic trochlea [21]

It is currently unknown what the ideal solution to each potential anatomic variation is, but a universal prosthetic position is unlikely to be the answer, especially in the circumstances of more extreme anatomy. Furthermore, not every individual native anatomy should be replicated by the implant positioning, since in specific cases with trochlear dysplasia and/or patellar instability, the anatomy itself was the reason for patellofemoral maltracking and development of arthritis.

A more biomimetic implant would ideally place the sulcus lateral to the condylar midpoint, align the sulcus more parallel to the sagittal plane, include an inflection point at the midpoint of the sulcus and restore lateral trochlear height. To do so, several variants with differing prosthetic DTSAs would be needed to accommodate variation in mLDFA. Alternatively custom made, patient specific implants or multicompartment unicompartmental replacements could be used. While these solutions would have obvious logistic difficulties, this could be addressed with appropriate preoperative planning. Furthermore, the cost effectiveness of these approaches needs to be proven, since they would be expected to -at least at the beginning- raise the implant costs.

Regarding the anatomical differences based on sex, while there is a good evidence base highlighting the differing distal femoral aspect ratios between men and women [22, 23] the same cannot be said for differing the prosthetic DTSA. This is unequivocally demonstrated in our results, suggesting that alterations of prosthetic DTSA should be based on mLDFA rather than sex. This is perhaps particularly the case in the context of KA where the argument could be made for standard and valgus mLDFA variants.

If the design and placement of femoral TKA components are to continue in a biomimetic direction changes will need to be made to trochlear design. For this to be achieved, the first prerequisite is a deep understanding of normal anatomy coupled with an understanding of the tolerances of polyethylene and bone cement. A single femoral component design and alignment is unlikely to accommodate the wide range of variations in human distal femoral anatomy.

## Strengths and limitations

The main strength of this study is the sample size, which exceeds by far any previous studies reporting on the trochlear anatomy. The large sample size allowed the examination of the trochlear anatomy in association with other anatomical parameters, such as mLDFA, as well as across sex, ethnicity, BMI. Strong correlations were demonstrated. Furthermore, the use of a validated imaging methodology and a standardized reference coordinate system allows for accurate measurements. The exclusion of arthritic knees could be a possible limitation. However, the position and orientation of the trochlea should not be influenced by the presence of arthritis. Furthermore, this study was based purely on CT measurements; hence, the results are dictated from the bony morphology which has been shown to differ from that of the overlying cartilage [24, 25]. However, the orientation of the trochlear sulcus, which was the main interest of this study, should not be significantly affected by the presence of cartilage [25].

## Conclusions

In non-arthritic knees, there is a strong positive correlation between the mLDFA and the DTSA. Consequently, the trochlear sulcus is consistently orientated in the sagittal plane regardless of distal condylar anatomy. The sulcus has a bilinear morphology with an inflection point at the midpoint of its arc. The LSA is essentially constant throughout the length of the sulcus. These findings have relevance to biomimetic prosthetic design and kinematic positioning of existing implants.

## Abbreviations

TKA: Total knee arthroplasty
KA: Kinematic alignment
MA: Mechanical alignment
mLDFA: Mechanical lateral distal femoral angle
CT: Computerized tomography
MSA: Medial sulcus angle
LSA: Lateral sulcus angle
DFJL: Distal femoral joint line
DTSA: Distal trochlear sulcus angle
HKAA: Hip knee ankle angle
BMI: Body mass index
